# A transcriptome map of cellular transformation by the fos oncogene

**DOI:** 10.1186/1476-4598-4-19

**Published:** 2005-05-26

**Authors:** Jared M Ordway, Steven D Fenster, Hong Ruan, Thomas Curran

**Affiliations:** 1 Department of Developmental Neurobiology, St. Jude Children's Research Hospital, 332 N. Lauderdale St., Memphis, TN, 38105, USA; 2 Orion Genomics, 4041 Forest Park Ave., St. Louis, MO, 63108, USA

## Abstract

**Background:**

The c-*fos *gene was originally identified as the cellular homolog of the oncogene v-*fos *carried by the Finkel-Biskis-Jenkins and Finkel-Biskis-Reilly murine osteogenic sarcoma retroviruses. Sustained expression of *fos *is sufficient to induce cellular transformation *in vitro *and tumorigenesis *in vivo*. Fos functions as a component of the AP-1 transcription factor complex to regulate gene transcription and several differentially expressed genes have been identified in cells transformed by *fos*. We have extended these studies by constructing a cellular system for conditional transformation by v-*fos*. Using Affymetrix-based DNA microarray technology, we analyzed transcriptional changes over the course of transformation and reversion in an inducible v-fos system.

**Results:**

Microarray analyses of temporal gene expression during the process of *v-fos *mediated cellular transformation and morphological reversion revealed a remarkably dynamic transcriptome. Of the more than 8000 genes analyzed in this study, 3766 genes were categorized into 18 gene-expression patterns by using self-organizing map analysis. By combining the analysis of gene expression profiles in stably transformed cells with the analysis of sequential expression patterns during conditional transformation, we identified a relatively small cohort of genes implicated in *v-fos *mediated cellular transformation.

**Conclusion:**

This approach defines a general conditional cell transformation system that can be used to study the endogenous transcription regulatory mechanisms involved in transformation and tumorigenesis. In addition, this study is the first reported analysis of dynamic changes in gene expression throughout experimentally controlled morphological transformation mediated by v-*fos*.

## Background

The c-*fos *proto-oncogene encodes an immediate-early transcription factor that is rapidly and transiently induced by a variety of extracellular stimuli associated with cellular responses such as proliferation, differentiation, apoptosis and neuronal signalling [1]. The c-Fos protein functions by forming leucine zipper dimers with members of the Jun and ATF/CREB families that comprise the transcription factor complexes collectively referred to as AP-1 [2]. The tightly regulated expression and activity of AP-1 family members defines a prototypical mechanism whereby short-term extracellular signals are coupled to appropriate long-term changes in cellular phenotype by selective regulation of gene expression.

The identification of v-*fos *as the oncogene carried by the Finkel-Biskis-Jenkins and Finkel-Biskis-Reilly murine osteosarcoma retroviruses contributed to the realization that tumorigenic retroviruses harbor viral versions of cellular genes and that these genes can elude the regulatory constraints imposed upon the endogenous gene [3-5]. The viral *fos *oncogenes contain point mutations and deletions that enhance their transforming potential [6]. However, sustained expression of c-*fos *is sufficient to induce cellular transformation *in vitro *and tumorigenesis *in vivo *[7]. Therefore, *fos*-induced transformation and tumorigenesis is the consequence of inappropriate *fos *activity within susceptible cells rather than a gain-of-function mechanism specific to the viral *fos *oncogene.

Many signal transduction pathways implicated in tumorigenesis functionally converge on activation of c-*fos *and AP-1, suggesting that inappropriate activation of c-*fos *contributes to various aspects of tumorigenesis. This contribution involves direct transcriptional regulation of AP-1 target genes and secondary mechanisms of transcriptional regulation. For example, increased expression and activity of *Dnmt1*, a DNA methyltransferase that methylates CpG dinucleotides [8], is necessary for morphological transformation by c-*fos *[9]. CpG methylation within promoter regions functions as an epigenetic mark that establishes or maintains transcriptional repression by recruiting chromatin modification machinery [10]. A previous study identified specific genes that are irreversibly silenced in association with DNA hypermethylation in *fos*-transformed cells [11]. Therefore, during *fos*-mediated transformation, there is conditional deregulation of target gene expression dependent upon continual oncogene activity, in addition to long-term epigenetic reprogramming of gene expression that can persist even when the direct effects of oncogene activity are suppressed.

Studies of stably transformed cell lines have found gene expression changes associated with *fos *transformation and have yielded functional data that implicate differentially expressed genes in aspects of oncogenic transformation [12-14]. In the study described here, we took advantage of a conditional cellular system (LacIv-*fos*) that allows control of v-*fos *expression and morphologic transformation. This approach refines the analysis of gene expression associated with *fos *transformation by distinguishing gene expression changes coincident with morphological transformation from those that are potentially associated with clonal variation or phenotypic changes that occur downstream of the transformation process. Comparisons of temporal gene expression patterns during conditional cellular transformation with transcriptome profiles of cells stably transformed by c-*fos *and v-*fos *revealed a cohort of genes likely to be critical for induction and maintenance of cellular transformation.

## Results

### Inducible lacIv-*fos *system

**Figure 1:**
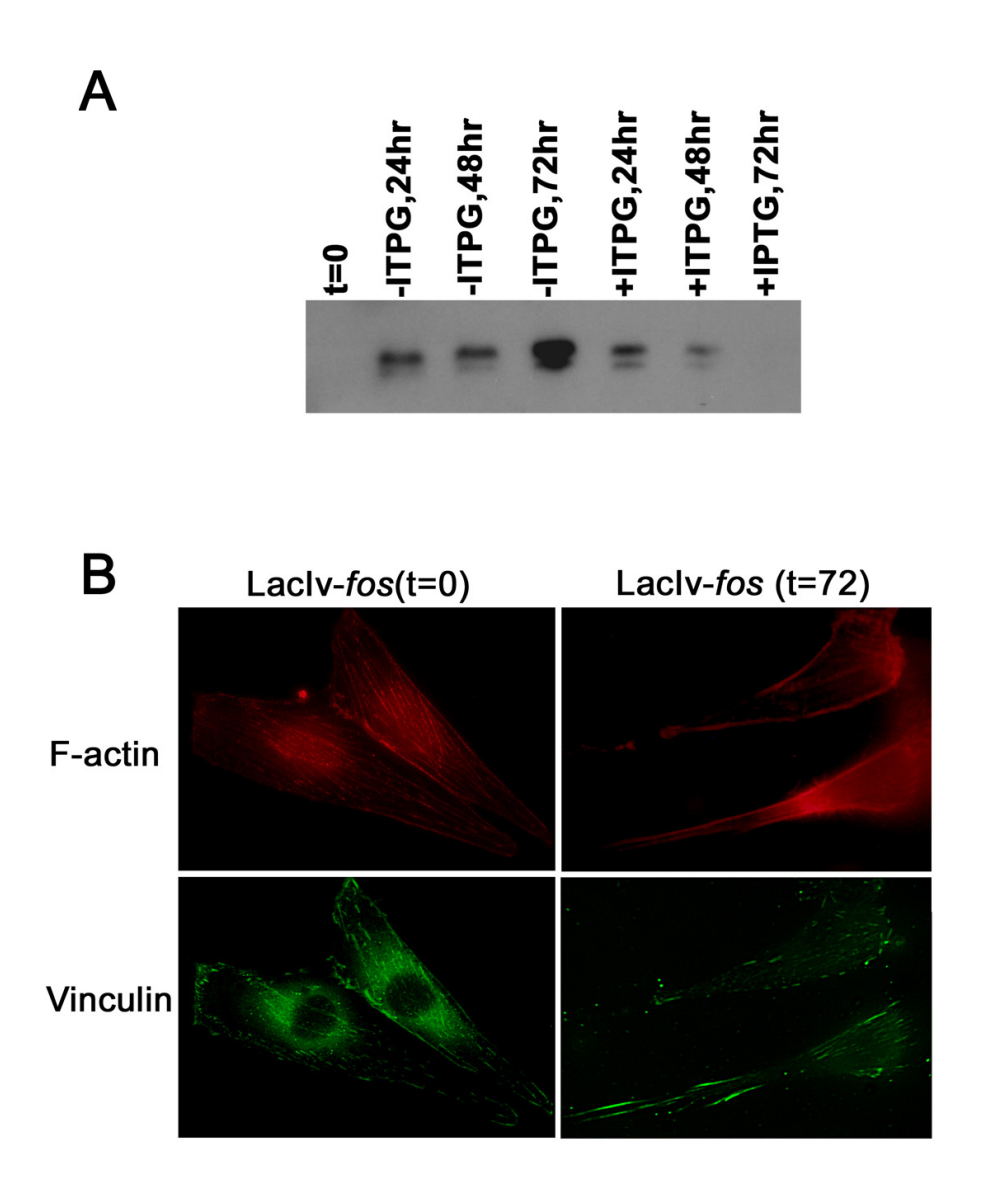
**Expression of v-Fos and cell morphology rearrangement in LacIv-*fos *system during conditional transformation and reversion**. (A) Expression of v-Fos protein expression during conditional morphological transformation of LacIv-*fos *cells. At time zero (t = 0), Fos protein was not detectable in cells. 24 hours after removal of IPTG, Fos protein expression was induced and the immunoreactivity increased over the 72 hour transformation period. After re-addition of IPTG, Fos protein levels rapidly decreased with no detectable signal observed after 72 hours of reversion. (B) Induction of v-Fos expression resulted in dramatic cytoskeletal changes in LacIv-*fos *cells. Cells were stained with anti-vinculin antibody (green) to detect focal adhesion contact sites and phalloidin (red) was used to track alterations in the actin cytoskeleton. In the presence of IPTG (t = 0), cells have well established focal adhesion sites and coordinated F-actin staining. Seventy-two hours after removal of IPTG (t = 72), cells lacked defined focal adhesion contacts and displayed disorganized F-actin staining

In the LacIv-*fos *cell system, the control of FBJ/R v-*fos *expression is dependent on the presence of isopropyl-b-D-thiogalactopryanoside (IPTG) in the cell culture medium [11]. In the presence of 5 mM IPTG, LacIv-*fos *cells did not express v-Fos protein detectable by Western blot analysis (Figure 1a). When IPTG was washed away, LacIv-*fos *cells expressed v-Fos protein with peak expression detected at 72 hours following removal of IPTG and progressive loss of v-Fos protein levels upon re-addition of IPTG. In addition, cells were morphologically transformed within a 72-hour period. Transformation was indicated by an overall change in cell shape that led to a more rounded and light refractory morphology as well as dramatic cytoskeletal alterations (Figure 1b). When IPTG was added back to these transformed cells, v-Fos expression was again repressed and the cells returned to their original morphology within a 72-hour reversion period.

### Self-organizing map analysis

The ability to control both v-Fos expression and morphological transformation *in vitro *provides an opportunity to investigate global gene expression changes relative to the temporal v-Fos cellular transformation and reversion process. RNA was extracted from LacIv-*fos *cells treated with 5 mM IPTG at time zero (t = 0); from cells at 24, 48 and 72 hours following the removal of IPTG (transforming); and from cells at 24, 48 and 72 hours after the re-addition of 5 mM IPTG (reverting). Previous studies have demonstrated that the addition and removal of IPTG does not itself induce persistent changes in gene expression in the parental 208F rat fibroblast cell line [9,11]. RNA samples were processed and analyzed by hybridization to Affymetrix rat U34A GeneChip microarrays.

**Figure 2:**
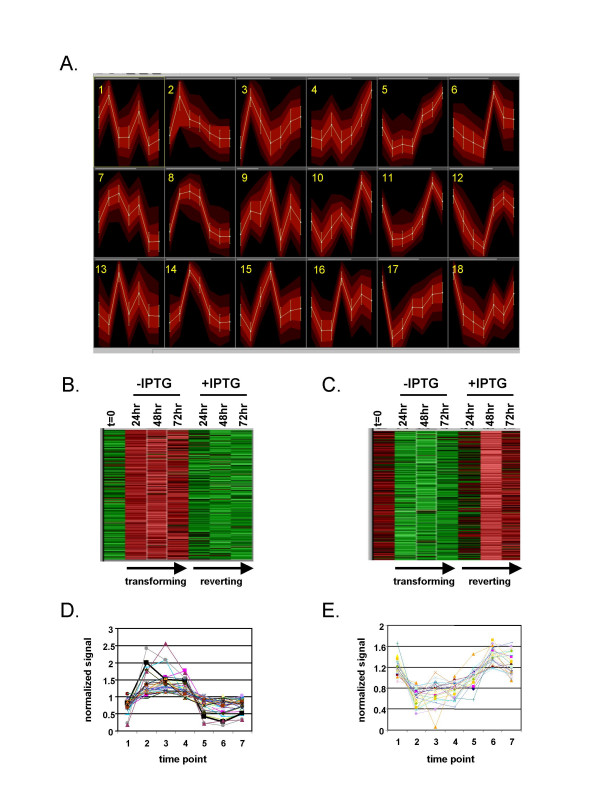
**Self-organizing map (SOM) analysis of microarray expression data in the LacIv-*fos *system during morphological transformation and reversion. **(A) Results of SOM analysis of genes depicting co-regulated clusters of genes across the seven time points throughout v-Fos mediated morphological transformation and reversion. Genes whose expression did not change significantly across time points were eliminated by using a variance filter (see Methods). The 3766 probe sets (out of a possible 8799) that passed the variation filter were grouped into 18 clustered patterns. Blue bars at the top of each graph represent the relative number of probe sets included within the SOM bin. (B) A gene-expression heat map of genes within group 8. This bin represents genes dramatically upregulated specifically during the 72 hour v-Fos transformation period (-IPTG). (C) Gene-expression heat map of genes within group 11. This bin represents genes displaying sustained downregulation during the 72 hr v-Fos transformation period (+ITPG). Red signal represents upregulated genes and green represents downregulated genes. (D-E) Normalized expression values of all genes within group 8 (D) or group 11 (E) are plotted to demonstrate the reliability of the representative SOM patterns shown in panel A.

Self-organizing map (SOM) analysis is a powerful tool that can be used to categorize gene expression data into groups that share common temporal expression profiles [15-17]. To identify patterns of gene expression, we created an SOM by using the LacIv-*fos *microarray data obtained at the seven time points throughout v-Fos transformation and reversion (Figure 2a). The data were mapped into 18 groups that provided the greatest pattern distinction among groups as well as the best pattern consistency within groups. Several assumptions were made on the basis of simple visualization of the map. For example, the expression of genes grouped into pattern 1 peaked 24 hours following removal of IPTG, as well as at 24 hours following re-addition of IPTG. This group likely represents genes whose expression was induced within 24 hours following culture media change rather than genes whose expression coincided with v-Fos-mediated transformation. Indeed, genes represented by pattern 1 included serum-responsive genes such as c-Jun and cyclin D1. However, other patterns included genes that were regulated in a manner more consistent with conditional v-Fos transformation and reversion. For example, pattern 8 included genes that were dramatically upregulated during the 3-day transformation process, yet return to baseline levels during the three-day reversion process (Figure 2b). This group included two probe sets specific for FBR v-*fos *included on the Affymetrix U34A GeneChip. These probe sets give a reliable measurement of FBJ/R v-*fos *expression and they did not cross-hybridize with endogenous c-*fos *transcripts [11]. Conversely, group 11 represents genes that were conditionally downregulated specifically during conditional v-Fos transformation (Figure 2c). Plots of the normalized signal values for each probe set included in these groups further confirmed the reliability of the classifications of gene expression profiles within the SOM (Figure 2d-e).

Patterns within the SOM that are consistent with v-Fos transformation and reversion were selected on the basis of the representative expression pattern of each SOM group. For candidate genes whose expression was upregulated, the standard deviation of the mean signal values at a minimum of two of the three time points in the absence of IPTG (transforming) had to be greater than that of the mean signal values at time zero (i.e., the time prior to removal of IPTG from the culture medium). Also, the standard deviation of the mean signal values at a minimum of two of the three final time points in the presence of IPTG (reverting) had to be below that of the mean signal value of the final time point during transformation (i.e., 72 hours after removal of IPTG from the culture medium). For candidate genes whose expression was downregulated, the standard deviation of the mean signal values at a minimum of two of the three transforming time points had to be lower than that of the mean signal values at time zero, and the standard deviation of the mean signal values at a minimum of two of the three time points during reversion had to be higher than that of the final time point during transformation. On the basis of these criteria, patterns 7, 8, 14 and 15 (transforming) and patterns 5, 11, 12, 17 and 18 (reverting) were selected for further analysis. Each of these groups consisted of a large number of genes (Figure 2a). These groups likely included not only genes functionally involved in cellular transformation, but also genes that share similar expression profiles during conditional transformation and reversion yet have no role in the process or maintenance of cellular transformation itself. Interestingly, both patterns 5 and 7 included genes with higher signal-ratio values at the end of morphological reversion (t = 72 hours, reverting) than prior to v-fos induction (t = 0). This difference in transcript levels at the beginning of the time course relative to the final time point may be related to a requirement for a higher level of expression of particular genes during the reversion of morphological transformation than during maintenance of the non-transformed state. Alternatively, this may reflect a type of over-compensation in that expression levels within particular expression pattern groups and transcript levels may not completely return to steady-state within the three-day reversion period.

### Stable versus inducible v-*fos *expression

To identify only the most promising candidate genes, we compared these datasets with gene expression profiles of the parental 208F cells and 208F cells transformed by stable expression of either c-Fos (CMVc-*fos *cells) or v-Fos (FBJ/R cells). Cells transformed by stable expression of c-Fos or v-Fos exhibit a large number of differentially expressed genes. For example, when differential expression was defined as a 2-fold increase or decrease based on a comparison with expression in 208F cells and when probe sets for expressed sequence tags (ESTs) were excluded, 70 probe sets were scored as upregulated and 104 were scored as downregulated in both CMVc-*fos *and FBJ/R cells [11]. However, eliminating genes whose expression changes are consistent in both stably and conditionally transformed cell model systems can reduce the level of nonspecific transcriptional variation. Of the 174 differentially expressed probe sets identified in comparisons of both CMVc-*fos *and FBJ/R cells to 208F cells, 63 (36%) were also categorized into LacIv-*fos *SOM patterns consistent with increased or decreased expression during conditional v-Fos transformation. Therefore, the majority of gene expression changes associated with stably Fos-transformed cell lines were not recapitulated during conditional v-Fos transformation and reversion. These findings emphasize the value of combining analyses of stable and conditional cellular transformation systems.

**Figure 3:**
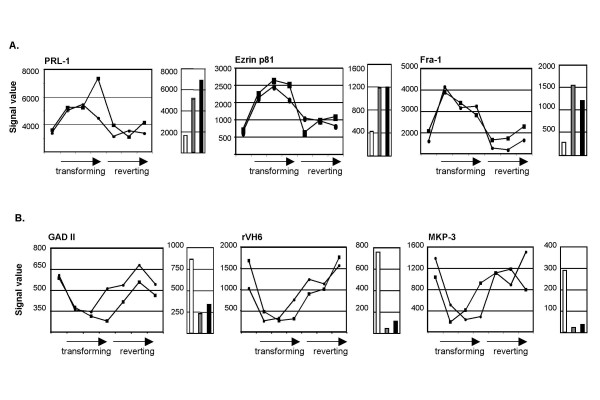
**Expression of representative genes conditionally regulated in LacIv-*fos *cells and differentially expressed in both CMVc-*fos *cells and FBJ/R cells**. Expression patterns of selected genes upregulated (A) or downregulated (B) specifically during conditional transformation, as well as in cells stably transformed by either c-Fos or v-Fos. Gene names are indicated above each pair of graphs. Line graphs indicate raw signal values obtained from the two independent LacIv-*fos *time course experiments. Time points at 24, 48 and 72 hours in the absence of IPTG (transforming) and at 24, 48 and 72 hours after the re-addition of IPTG (reverting) are indicated by arrows below each line graph. Bar graphs indicate raw signal values obtained in microarray analyses of the parental 208F fibroblast cell line (white), the stably transformed c-*fos *cell line (CMVc-*fos*; grey) and the stably transformed v-*fos *cell line (FBJ/R; black).

To address the reproducibility of gene expression changes during conditional v-Fos transformation, we performed microarray analysis using RNA samples isolated from LacIv-*fos *cells at time points during a second LacIv-*fos *transformation and reversion time course experiment. To maintain stringent biological replication of the experiment, these seven time points were treated as an independent experiment rather than as expression values that could be averaged between experiments. Expression patterns of genes differentially expressed in both CMVc-*fos *and FBJ/R cells relative to 208F cells, as well as conditionally regulated within the LacIv-*fos *SOM obtained from the first experiment, were compared to expression patterns obtained from the independent LacIv-*fos *transformation and reversion time course experiment. Of the 63 probe sets representing differentially expression in stably Fos-transformed cells and conditional regulation in the LacIv-*fos *SOM, 40 (64%) demonstrated a reproducible temporal profile of gene expression in the independent LacIv-*fos *transformation and reversion time course experiment (Additional file 1 and Figure 3). In addition, a previous study indicated by Northern blot analysis that representative genes (i.e. *TGF-β *3 and *CAIII*) had nearly identical patterns of gene expression when compared to microarray analysis [11]. Therefore, the combination of gene expression analysis of stably transformed cells with the analysis of gene expression in an experimentally controlled conditional transformation system is a useful approach to reduce complicating transcriptional variation inherent in any single cell model system.

### *c-fos *versus *v-fos *gene regulation

**Figure 4:**
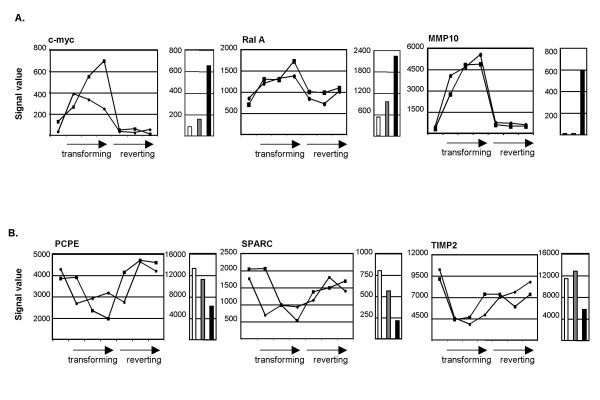
**Expression of representative genes conditionally regulated in LacIv-*fos *cells and stably regulated in FBJ/R cells, but not CMVc-*fos *cells. **Expression patterns of selected genes upregulated (A) or downregulated (B) specifically during conditional transformation, as well as in cells stably transformed by v-Fos. Gene names are indicated above each pair of graphs. Line graphs indicate raw signal values obtained from the two independent LacIv-*fos *time course experiments. Time points at 24, 48 and 72 hours in the absence of IPTG (transforming) and at 24, 48 and 72 hours after the re-addition of IPTG (reverting) are indicated by arrows below each line graph. Bar graphs indicate raw signal values obtained in microarray analyses of the parental 208F fibroblast cell line (white), the stably transformed c-Fos cell line (CMVc-*fos*; grey) and the stably transformed v-Fos cell line (FBJ/R; black).

Unlike c-*fos*, v-*fos *contains several deletions and point mutations that affect its oncogenic potential [18]. Although sustained expression of c-*fos *is capable of inducing cellular transformation and tumorigenesis, v-*fos *is a much more potent oncogene [7]. The increased tumorigenic potential of v-Fos suggests that it directly or indirectly influences the expression of genes not affected by c-Fos. Consistent with this hypothesis, gene expression profile analyses to identify genes differentially expressed by a 2-fold margin in FBJ/R cells, but not in CMVc-*fos *cells, revealed a dramatic increase in the number of transcriptionally upregulated genes in FBJ/R relative to CMVc-*fos *cells: 337 probe sets were scored as specifically upregulated in FBJ/R cells, whereas only 70 probe sets common to CMVc-*fos *and FBJ/R cells were considered as increased. In contrast, 61 probe sets were scored as downregulated specifically in FBJ/R cells but 104 probe sets in both CMVc-*fos *and FBJ/R cells were considered as downregulated [11]. These FBJ/R cell-specific changes in gene expression were compared with the LacIv-*fos *SOM and the results of the independent LacIv-*fos *transformation and reversion time course as described previously. These comparisons revealed an additional 38 upregulated and 29 downregulated probe sets that are differentially regulated in v-*fos*-transformed cells, but not in c-*fos *transformed cells (Tables 1 and 2, Figure 4).

**Table 1:** Genes whose expression was upregulated (i.e., increased by a factor≥ 2) in FBJ/R cells (but not in CMVc-*fos *cells) and was conditionally regulated in LacIv-*fos *cells.

Upregulated v-Fos specific candidate genes		
GenBank Accession No.	Gene	Pattern

AF100470	ribosome attached membrane protein 4 (RAMP4)	7
AF033027	prenylated SNARE protein Ykt6p (Ykt6)	7
AF036537	homocysteine respondent protein HCYP2	7
AF054618	cortactin isoform C	7
D00092	mRNA for 70 kd mitochondrial autoantigen	7
D30740	14-3-3 protein mRNA for mitochondrial import stimulation factor (MSF) S1 subunit	7
M21476	iodothyronine 5-monodeiodinase (5-MD)	7
M96630	sec61 homologue	7
U49930	ICE-like cysteine protease, Lice (aka Caspase 3)	7
U95161	nuclear protein E3-3 orf2	7
X13722	LDL-receptor	7
X70871	cyclin G	7
X92097	transmembrane protein rnp21.4	7
Y09332	cytosolic peroxisome proliferator-induced acyl-CoA thioesterase	7
AB015432	LAT1 (L-type amino acid transporter 1	8
AF069782	unknown mRNA (aka NAP65)	8
D16479	mitochondrial long-chain 3-ketoacyl-CoA thiolase beta-subunit of mitochondrial trifunctional protein	8
D26393	HK2 gene for type II hexokinase*	8
L12025	tumor-associated glycoprotein E4 (Tage4)	8
L12382	ADP-ribosylation factor 3	8
L19698	GTP-binding protein (ral A)	8
L38644	karyopherin beta	8
M62992	glycoprotein p62	8
U21718	clone C426 intestinal epithelium proliferating cell-associated mRNA	8
U38253	initiation factor eIF-2B gamma subunit (eIF-2B gamma)	8
X82445	C15 mRNA	8
Y00396	c-myc oncogene	8
M58587	interleukin 6 receptor ligand binding chain	14
M65253	transformation-associated protein, 34A (aka MMP10)	14
U17901	phospholipase A-2-activating protein (plap)	14
U35774	cytosolic branch chain aminotransferase 1, cytosolic	14
U92081	epithelial cell transmembrane protein antigen precursor (RTI40)	14
D12498	FGF receptor-1*	15
M74223	VGF mRNA	15
S54008	fibroblast growth factor receptor 1 beta-isoform*	15
S56464	hexokinase II (HK2)*	15
S81025	UDP-galactose:N-acetylglucosamine beta-1,4-galactosyltransferase homolog	15
J05166	band 3 Cl-/HCO3- exchanger, B3RP2 (aka Slc4a2)	15

**Table 2:** Genes whose expression was downregulated (i.e., decreased by a factor ≥ 2) in FBJ/R cells (but not in CMVc-*fos *cells) and was conditionally regulated in LacIv-*fos *cells.

Downregulated v-Fos-specific candidate genes		
GenBank Accession No.	Gene	Pattern

M64780	agrin	5
M38135	cathepsin H (RCHII)	11
U52663	peptidylglycine alpha-amidating monooxygenase (PAM)	11
U75917	clathrin-associated protein 17 (AP17)	11
U75929	SPARC (aka osteonectin)*	11
X05341	3-oxoacyl-CoA thiolase	11
D10026	glutathione S-transferase Yrs-Yrs	12
J02791	acyl coenzyme A dehydrogenase medium chain	12
J03752	glutathione S-transferase	12
M93257	cathechol-O-methyltransferase	12
X05472	Rat 2.4 kb repeat DNA right terminal region	12
X74593	sorbitol dehydrogenase	12
Y09333	mitochondrial very-long-chain acyl-CoA thioesterase	12
AF034218	hyaluronidase (Hyal2)	17
AF065387	vitamin K-dependent gamma-glutamyl carboxylase	17
J02810	prostate glutathione S-transferase	17
J05031	isovaleryl-CoA dehydrogenase (IVD)	17
L01702	protein-tryosine-phosphatase (LRP)	17
U10357	pyruvate dehydrogenase kinase 2 subunit p45 (PDK2)	17
U25651	phosphofructokinase muscle isozyme	17
U75928	SPARC (aka osteonectin)*	17
Y13714	osteonectin (aka SPARC)*	17
D00512	mitochondrial acetoacetyl-CoA thiolase precursor	18
D13921	mitochondrial acetoacetyl-CoA thiolase	18
D16309	cyclin D3	18
M60921	NGF-inducible anti-proliferative putative secreted protein (PC3)	18
S7259	tissue inhibitor of metalloproteinase type 2	18
X95986	CBR gene	18

### LacIv-*fos *web-accessible database

The identification of conditionally regulated genes within this system provides potentially unique opportunities to investigate precise transcriptional regulatory mechanisms of endogenous genes within an experimentally amenable cellular context. Therefore, we assembled these datasets into a web-accessible database equipped with various query functions so that users can mine the data to address questions relevant to their own specific interests (see methods). The initial interface of the database is the LacIv-*fos *SOM (Figure 2a). Users can select SOM patterns of interest to obtain a list of all probe sets mapped within that common pattern of gene expression. Each probe set is linked to the raw Affymetrix data for both independent LacIv-*fos *time course experiments. This arrangement provides an opportunity for convenient confirmation of consistent regulation between the independent time course experiments. In addition, users can query the database for information about specific genes by using Genbank accession numbers or by using genes name as keywords. Keyword queries may include the qualifier functions "and" or "or". These queries will provide a list of all probe sets matching the supplied criteria, and each entry is again linked to the raw Affymetrix data obtained from both independent LacIv-*fos *time course experiments.

## Discussion

The process of cellular transformation involves complex alterations of gene expression regulation. This level of complexity raises the challenge of identifying the gene expression changes that are most relevant to mechanisms of tumorigenesis. The challenge is especially evident in studies of primary tumor specimens in which the analyzed material represents the end state of a progressive series of events rather than a snapshot of the process itself. Cell-based model systems of transformation and tumorigenesis offer an opportunity to experimentally simulate steps along the pathway of transformation; however, these systems are subject to their own set of caveats including gene expression changes resulting from variation inherent to cell culture conditions and clonal variation. In the study described here, we attempted to provide a more focused view of relevant gene expression changes associated with oncogenic cellular transformation by analyzing a conditional cell-based model system in which morphological transformation and reversion is experimentally manipulated.

Sustained expression of *fos *results in the differential expression of a large number of genes [7,9,11]. By analyzing gene expression at time points throughout the conditional cellular transformation and reversion of LacIv-*fos *cells and by arranging these patterns into SOMs, we have clustered differentially expressed genes into cohorts with common temporal patterns of expression. This type of clustering allows identification of groups of genes with expression patterns that are consistent with a potential functional role in the process of morphological transformation. For example, the exogenous v-*fos *transcript itself is clustered within the conditionally upregulated group 8. This group also includes the previously reported Fos-regulated target genes such as *ezrin *[19] and *Fra-1 *[20,21] genes shown to be strongly upregulated in response to fos-induced transformation [19,21]. Interestingly, independent genes with functionally related roles in cellular transformation fall into different expression pattern bins. For example, ezrin and CD44 form a complex that plays an active role in aspects of tumor progression and metastasis such as tumor-endothelium interactions, cell migration and cell adhesion [22] but the expression patterns of these two genes differ somewhat. The expression of *CD44 *is more transiently induced (group 7); this characteristic is consistent with features of a gene whose expression is induced in response to serum stimulation. In contrast, *ezrin *expression is maintained at its maximal level throughout the 3-day morphological transformation period and rapidly returns to its initial expression levels upon repression of exogenous v-*fos *expression (group 8). These results demonstrate that coordinated yet distinct programs of gene expression regulation can be initiated to affect the expression of genes that function in common mechanisms of cellular transformation.

Our analysis has revealed additional conditionally regulated genes that have not been previously associated directly with Fos or AP-1. For example, PRL-1 is a protein tyrosine phosphatase originally identified as an immediate-early gene in liver regeneration [23]. PRL-1 expression is elevated in cells stably transformed by either c-*fos *or v-*fos*, and it is conditionally upregulated during LacIv-*fos *transformation (group 8). PRL-1 promotes cell migration and invasion *in vitro *and its expression is elevated in a number of cancer cell lines. PRL-1 is also involved in regulation of progression through mitosis, possibly by modulating spindle dynamics [24,25].

Sustained expression of c-Fos is capable of initiating transformation [7]; however, in our study a comparison of c-Fos transformed cells (CMVc-*fos*) with v-Fos transformed cells revealed additional differential gene regulation events in cells transformed by v-Fos relative to cells transformed by c-Fos. For example, expression signal values of both c-*myc *and the Ras GTPase, *RalA*, were slightly greater in CMVc-*fos *cells relative to 208F cells. However, expression of these genes was dramatically upregulated (>2fold) in FBJ/R cells. Likewise, *PCPE *and *SPARC *expression was slightly downregulated in CMVc-*fos *cells, but were dramatically repressed in FBJ/R cells. In contrast, two genes involved in enzymatic regulation of the extracellular matrix were differentially expressed only in FBJ/R cells. Expression of the matrix metalloproteinase *MMP10 *and the metalloproteinase inhibitor *TIMP2 *was specifically upregulated and downregulated in v-*fos*-transformed cells, respectively. Interestingly, *MMP10 *was previously identified as a gene whose expression was upregulated gene in cells stably transformed by FBR-v-*fos *[14]. The mechanisms responsible for differential expression of these genes specifically in v-*fos*-transformed cells are currently unknown. Some of these genes may be direct targets of v-Fos, but not of c-Fos, or targets of secondary transcription regulatory factors whose expression is deregulated specifically in v-*fos*-transformed cells. Alternatively, the expression of some genes may be affected by both c-Fos and v-Fos, but to a higher degree in v-*fos*-transformed cells.

The identification of 28 conditionally downregulated probe sets specific to v-Fos transformation raises the possibility that loss of expression of a subset of these genes may be due to chromatin modifying mechanisms leading to gene silencing. Included in our analysis are several genes previously implicated as targets for gene silencing via epigenetic repression. For example, the extracellular matrix molecule, SPARC, is downregulated in c-*jun*-transformed primary rat embryo fibroblasts [26]. Also, in v-j*un*-transformed chick embryo fibroblasts, reduction in SPARC mRNA levels has been shown to be due in part to the formation of a DNA-Sp1/3-v-Jun chromatin-associated complex [27]. Tissue inhibitor of metalloprotease 2 (TIMP-2), an endogenous inhibitor of MMP-2, has been shown to inhibit invasion and metastasis [28] and overexpression of TIMP-2 inhibited growth and reduced invasive potential in tumor cells [29]. TIMP-2 is subject to aberrant promoter hypermethylation in human cervical cancer cells and increased methylation favors development of primary cervical cancers [30]. Interestingly, treatment of human neuroblastoma cells with the DNA methyltransferase inhibitor 5-azacytidine (5-AzaC) restored TIMP-2 expression and resulted in a reduction of *in vitro *invasiveness [31]. The precise role of *v-fos *in mediating gene silencing in the conditional system is not clear; however, our system represents a tool for identifying candidate genes that are subject to epigenetic modification during the process of oncogenesis, as well as a conditional cellular system that can be employed to investigate the temporal mechanisms underlying these regulatory events.

## Conclusion

Technological advances in the ability to analyze gene expression profiles on an increasingly global scale have contributed significantly to a more comprehensive view of the complex transcriptional networks that go awry in tumor cells [32,33]. Studies of *in vitro *cellular model systems of oncogenic transformation have provided a wealth of information relevant to both normal signal transduction pathways and tumorigenic mechanisms. However, the large number of genes differentially expressed in these model systems often complicates the identification of the most promising candidate genes for further study. Our web-accessible database of transcriptional changes detected in the conditional v-fos system provides a powerful tool to identify cohorts of gene candidates associated with specific cellular events during the process of transformation and reversion.

## Methods

### Cell culture and v-*fos *transformation and reversion time course

All cells were maintained in DMEM supplemented with 10% fetal calf serum, L-glutamine and penicillin/streptomycin at 37°C in the presence of 5% CO_2_. Establishment of the stably transformed cell lines, FBJ/R and CMVc-*fos*, was described previously [9]. Establishment of the LacIv-*fos *conditional cell line and the conditions required for the transformation/reversion time course were described previously [11]. Briefly, for the v-*fos *transformation and reversion time course experiment, total RNA was extracted from LacIv-fos cells cultured in the presence of 5 mM IPTG at time zero; 24, 48 and 72 hours after removal of IPTG (transforming); and 24, 48 and 72 hours after the re-addition of 5 mM IPTG (reverting). Total RNA was extracted from cells by using Tripure reagent according to the manufacturer's instructions (Roche). The integrity of all RNA samples was verified by using an Agilent 2100 Bioanalyzer.

### Western blot and immunofluorescence analyses

Expression of Fos protein during transformation and reversion was evaluated by Western blotting analysis using an antibody directed against Fos [7]. Whole-cell lysates were prepared in lysis buffer (20 mM Tris pH 7.5, 100 mM NaCl, 0.5% NP-40, protease inhibitor cocktail [Roche]) and separated on a 12% SDS-polyacrylamide gel. For immunofluorescence microscopy, cells were fixed in 4% (w/v) formaldehyde. Fixed cells were incubated with primary mouse monoclonal anti-vinculin antibody (Sigma; dilution of 1:200) and phalloidin-congjugated to Alexa-568 (Molecular Probes). An anti-mouse secondary antibody (dilution of 1:1000) conjugated to Alexa-488 (Molecular Probes) was used to detect vinculin.

### Microarray analyses

RNA samples were processed for hybridization without amplification and hybridized to Affymetrix Rat Genome U34A GeneChips that include probe sets representing approximately 7,000 annotated genes and 1,000 EST clones [34]. Labelling and hybridization were preformed as described [35]. GeneChips were scanned using a laser confocal scanner (Agilent Technologies) and images were analyzed using Affymetrix Microarray Suite v.5.0. Datasets were standardized by global scaling of the average fluorescent intensities of all probe sets to a constant target value of 500 for all arrays. Quality control parameters for each hybridization were within MIAME compliant specifications [36]. A variance filter was applied to the dataset to remove data from probe sets representing genes that were not expressed throughout the time course and to standardize expression values for genes whose expression was scored absent at particular time points [37]. Data that were derived from probe sets that reported absent change calls at all seven time points were removed from the analysis. To standardize expression values of genes whose expression was scored as absent at fewer than seven time points, we converted the signal values that corresponded to absent change calls to a value of 1. Genes scored as differentially expressed in CMVc-*fos *cells and FBJ/R cells relative to 208F cells had a signal log ratio ≥ 1 (increased) or ≤ -1 (decreased), and a change p-value < 0.001 (increased) or > 0.99 (decreased). For visualization of specific profiles of gene expression during the LacIv-fos conditional transformation and reversion time course, signal values obtained at each LacIv-fos time point were plotted by using Microsoft Excel.

### SOM analysis

Microarray data that were obtained from the LacIv-fos time course experiments and passed the variance filter were grouped into relative expression pattern bins by the self-organizing map (SOM) program in the GeneMaths software package (version 1.5; Applied Maths, Austin, TX). The SOM analysis was performed by using a 6 × 3 node format to allow optimal representation of gene expression patterns in a reasonably small number of independent bins.

### The SOM searchable database

The SOM searchable database has been implemented on an Open Source MySQL 4.0.14 relational database management system. The database has a web interface at . The web application to query and manage the database is driven with a server-side scripting technology JSP. The entries of this database are generated from the tab-delimited output files of the SOM analysis. Data obtained from the SOM analysis can be queried by three approaches. Users can click on a graphical pattern key to view a list of all genes whose expression profiles match the particular self-organization pattern. Each entry in the list contains two parameters associated with the respective gene probe, i.e., the Affymetrix probe set ID number and a description of the gene. The detailed Affymetrix analysis data for a probe set can be obtained by clicking the link of the respective probe set ID number. The corresponding Affymetrix analysis page provides information about the number of oligonucleotide pairs, the signal magnitude, the detection status, and the detection p value at seven different time points derived from 2 independent experiments. Another search method is to query via the GenBank accession number. The query by accession number is based on the fact that each Affymetrix probe set ID number in our current experiments corresponds to a gene accession number. If an accession number in a query matches with any part within a probe set ID number, the entry containing the probe set ID number, its SOM pattern, and gene description is displayed. Hyperlinks are provided for both probe set ID numbers and SOM pattern. By following the link for a probe set ID number, the user can view the detailed Affymetrix analysis data for a selected gene probe; by following the link for an SOM pattern, the user can view the detailed Affymetrix analysis data for all the gene probes within the selected pattern. Also, a keyword-based text search is included in the database. A search for keywords in the paragraph of the gene description field is conducted in a case-insensitive manner. The search-by-keyword method is facilitated by the use of Boolean operations. The search can narrow or broaden quickly the results of the search by combining two or more keyword(s) with only one type of Boolean (and / or) operation.

## Authors' contributions

JMO prepared cells, purified RNA, and analyzed the microarray data, SDF performed immunofluoresence microscopy and Western blot analysis, and HR designed the web-accessible database. JMO, SDF, HR, and TC composed the manuscript.

## Supplementary Material

Additional File 1 Genes differentially expressed (i.e., expression varied by a factor of at least 2) in CMVc-*fos *cells and in FBJ/R cells and conditionally regulated in LacIv-*fos *cells. Click here for file
