# Supplementary material for: A transcriptome map of cellular transformation by the fos oncogene
Source: Mol Cancer. 2005 May 26;4:19. doi: 10.1186/1476-4598-4-19 (PMC1177986; doi:10.1186/1476-4598-4-19)
Supplement: Additional File 1 — Genes differentially expressed (i.e., expression varied by a factor of at least 2) in CMVc-fos cells and in FBJ/R cells and conditionally regulated in LacIv-fos cells. [file 1476-4598-4-19-S1.doc]

| Table 1. Genes that were differentially expressed (i.e., expression varied by a factor of at least 2) in CMVc-*fos* cells and in FBJ/R cells and were conditionally regulated in LacIv-*fos* cells. | | | | | |
| --- | --- | --- | --- | --- | --- |
| Upregulated candidate genes | | | Downregulated candidate genes | | |
| GenBank Accession No. | Gene | Pattern | GenBank Accession No. | Gene | Pattern |
|  |  |  |  |  |  |
| M61875 | glycoprotein CD44 | 7 | AF015304 | equilibrative nitrobenzylthioinosine-sensitive nucleoside transporter (aka Slc29a1) | 11 |
| M64301 | extracellular signal-related kinase (ERK3) | 7 | AF063102 | calcium-independent alpha-latrotoxin receptor homolog 2 (CIRL-2)* | 11 |
| AF048687 | UDP-Gal:glucosylceramide beta-1,4-galactosyltransferase | 8 | S81497 | lysosomal acid lipase=intracellular hydrolase | 11 |
| L27843 | tyrosine phosphatase (PRL-1) | 8 | X14323 | IgG receptor FcRn large subunit p51 | 11 |
| M19651 | fos-related antigen (Fra-1) | 8 | AF051561 | Na-K-Cl cotransporter, Nkcc1 (aka Slc12a2) | 12 |
| U79417 | 71 kDa component of rsec6/8 secretory complex | 8 | J02592 | glutathione S-transferase Y-b subunit* | 12 |
| X03347 | FBR-murine osteosarcoma provirus genome | 8 | U03491 | transforming growth factor beta-3 | 12 |
| X62528 | ribonuclease inhibitor | 8 | X59864 | ASM15 (aka H19) | 12 |
| X67788 | ezrin p81 | 8 | E13557 | GADII* | 17 |
| X71898 | urinary plasminogen activator receptor 1 | 8 | J03914 | glutathione S-transferase Yb subunit* | 17 |
| D28557 | RYB-a | 14 | U72620 | Lot1 (aka Pleimorphic adenoma gene-like 1, Plagl1) | 17 |
| D38222 | protein tyrosine phosphatase-like protein | 14 | X06801 | vascular alpha-actin | 17 |
| D90109 | long-chain acyl-CoA synthetase | 14 | U42627 | dual-specificity protein tyrosine phosphatase, rVH6 (aka MKP-3)* | 18 |
| E12625 | novel protein which is expressed with nerve injury (aka sterol-C4-methyl oxidase-like, sc4mol) | 14 | U70825 | 5-oxo-L-prolinase | 18 |
| U16655 | phospholipase C delta-4 | 14 | X94185 | for dual specificity phosphatase, MKP-3* | 18 |
| X62875 | High Mobility Group Protein I (Y) | 14 | D88250 | serine protease | 5 |
| M63282 | leucine zipper protein (aka ATF3) | 15 | X59267 | drebrin A | 5 |
|  |  |  | M25073 | kidney Zn-peptidase aminopeptidase N | 5 |
|  |  |  | M64755 | cysteine sulfinic acid decarboxylase (aka GADII)* | 5 |
|  |  |  | M60616 | collagenase (UMRCase) | 5 |
|  |  |  | M26125 | epoxide hydrolase | 5 |
|  |  |  | AF076619 | molecular adapter rGrb14 (Grb14) | 5 |
|  |  |  | AJ005394 | collagen alpha 1 type V | 5 |
|  |  |  | AF081148 | CL2AA* (aka CIRL-2) | 5 |
